# Effects of collagen and chondroitin sulfate on relaxation at multiple magnetic field strengths

**DOI:** 10.1016/j.heliyon.2025.e41854

**Published:** 2025-01-13

**Authors:** Olli-Pekka Aro, Victor Casula, Nina E. Hänninen, Jouni Karjalainen, Mikko J. Nissi, Miika T. Nieminen, Henning Henschel

**Affiliations:** a Research Unit of Health Sciences and Technology, University of Oulu, Oulu, Finland; b Department of Technical Physics, University of Eastern Finland, Kuopio, Finland; c Department of Diagnostic Radiology, Oulu University Hospital, Oulu, Finland; d Department of Medicinal Chemistry, Uppsala University, Husargatan 3, Box 574, 75123, Uppsala, Sweden

**Keywords:** Chondroitin sulfate, Collagen, Cartilage, Gel phantom, Relaxivity

## Abstract

**Purpose:**

To elucidate the connection between MRI relaxation properties of articular cartilage and tissue composition, in terms of collagen and chondroitin sulfate (CS). Additional aims were to determine the effect of different magnetic field strengths, as well as the effect of concentrations of the components on relaxation properties.

**Methods:**

A series of MRI phantoms consisting of gels containing collagen and chondroitin sulfate were prepared with final concentrations of collagen in the range 20-60 mg/g and the CS concentration in the range 0-40 mg/g. $R_{1}$ (= 1/$T_{1}$) and $R_{2}$ (= 1/$T_{2}$) values of the phantoms were measured at three different MRI field strengths (1.5, 3.0 and 9.4 T), $R_{1\rho }$ (= 1/$T_{1\rho }$) values were measured at 9.4 T.

**Results:**

Relaxation rates generally increased with increasing concentration of either of the compounds. $R_{1}$ values generally increased with CS, and at clinically used magnetic fields, namely 1.5 T and 3.0 T, with collagen concentration. At 9.4 T, $R_{2}$ values also showed an increase with collagen concentration that could not be clearly identified at lower field strengths. $R_{1\rho }$ values increased with both collagen and CS concentration but the amplitude of the spin-lock pulse only had a limited effect on relaxation rates above 100 Hz.

**Conclusions:**

Our results suggest that $R_{1}$, $R_{2}$, and $R_{1\rho }$ are modulated by collagen and CS concentrations, with collagen likely dominating at physiological concentrations.

## Highlights

• Relaxation rates of collagen and chondroitin sulfate phantoms at 3 field strenghts.
• Collagen likely dominating relaxation rates at physiological concentrations.
• Linear extrapolation to physiological concentrations gives realistic relaxation rates.

## 1 Introduction

Conventional MR imaging uses $T_{1}$ and $T_{2}$ relaxation to provide contrast. One central problem with using $T_{1}$ and $T_{2}$ in diagnostics of articular cartilage is the establishment of well-defined MRI biomarkers for disease progression. [1] $T_{1\rho }$ relaxation, i.e. longitudinal relaxation in the rotating frame, is considered a promising tool in diagnostics. $T_{1\rho }$ measurements at low spin-lock frequencies are especially adept at measuring low-frequency molecular interactions [2], as between water and large molecules within the extracellular matrix (ECM) [3]. Changes in the ECM, such as increased water molecule movement [4], lead to increases in $T_{1\rho }$ values. [5]

Correlation between collagen and/or proteoglycan (PG) concentration and $T_{2}$ values is still disputed. Some studies have found a connection between $T_{2}$ and collagen [6], while others found that, compared to $T_{1\rho }$, $T_{2}$ correlated poorly with PG content in bovine cartilage. [5] $T_{1\rho }$ is generally dependent on the amplitude of the spin-lock pulse i.e. contains dispersion. The underlying effect causing this dispersion in cartilage is still unclear. Mlynárik et al. [7], [7] measured $R_{1\rho }$ (= 1/$T_{1\rho }$) using spin-lock frequencies of 0-2500 Hz at 2.95 T and 0-1000 Hz at 7.05 T and assigned dipolar interactions between water protons and oriented collagen fibers to be the main mechanism contributing to $R_{1\rho }$ values. They also concluded chemical exchange to be unlikely as cause of the correlation between $T_{1\rho }$ and PG content in cartilage.

Although previous studies have shown $T_{1}$ and $T_{2}$ to be correlated to water content, [8] few studies concerning the relaxation properties of collagen gels have been conducted. One of the earliest studies of the subject investigated the relaxation properties of native and denatured collagen gels at 0.7 T, finding that both $T_{1}$ and $T_{2}$ values decreased compared to pure water in low concentration collagen gels, with only $T_{2}$ depending on the structure of the gel. [9] The study also found $T_{1}$ and $T_{2}$ of denatured gels to decrease as collagen concentration increased. Edzes and Samulski [10], [10] showed that $R_{1}$ (= 1/$T_{1}$) values of hydrated collagen are mainly influenced by the macromolecular spin-lattice relaxation as a result of dipolar coupling between water and macromolecular protons. Watanabe et al. [11], [11] found both $T_{1}$ and $T_{2}$ values to decrease with increasing collagen concentration in collagen gels with added contrast agents. Takeuchi et al. [12], [12], [13] made a collagen gel sample with magnetically oriented collagen fibers and compared the $T_{2}$ relaxation time of the sample to a sample with randomly oriented fibers. They found that the $T_{2}$ was significantly shorter in the ordered sample. When oriented at approximately 55^∘^ from the main magnetic field i.e. close to the magic angle, samples showed increasing $T_{2}$ times at all concentrations. Kudo et al. [14], [14] studied the connection states of collagen gels using $T_{1}$ and $T_{2}$ as evaluation parameters, among others. In some of the gels collagen was cross-linked with glutaraldehyde, which they found to affect the $T_{2}$ values proportionally to the glutaraldehyde concentration, while leaving $T_{1}$ unaffected.

In this study, we aim to increase understanding molecular contributions to relaxation, by studying imaging phantoms containing collagen and chondroitin sulfate (CS) at concentrations closer to those found in cartilage than has been done in previous studies. This is done by developing a description of the sensitivity of the phantoms' relaxation properties, namely $R_{1}$, $R_{2}$ (= 1/$T_{2}$) and $R_{1\rho }$, to changes in concentrations of the constituents. Furthermore, we demonstrate how different magnetic field strengths, and, in $R_{1\rho }$ experiments, varying the spin-lock frequency, affect the relaxation rates.

## 2 Methods

### 2.1 Phantom preparation

Three series of hydrogel phantoms were prepared with final nominal collagen concentrations of 20, 40, and 60 mg/g, and nominal CS concentrations were ranging from 0 to 40 mg/g. Exact final concentrations of the samples are shown in Table 1.

**Table 1:** Weights of the samples after drying and exact collagen and CS concentrations of the samples.

Sample no.	Final Weight	Collagen	CS
	[g]	[mg/g]	[mg/g]
1	1.48	20.27	0
2	1.50	20	10
3	1.52	19.74	19.74
4	1.51	19.87	39.74
5	1.48	40.54	0
6	1.53	39.22	9.80
7	1.51	39.74	19.87
8	1.49	40.27	40.27
9	1.50	60	0
10	1.53	58.82	9.80
11	1.51	59.60	19.86
12	1.37	65.69	43.80

The initial gels were prepared based on a protocol by ibidi GmbH (ibidi, Gräfelfing, Germany) [15]. Chondroitin sulfate powder (from bovine trachea, Merck KGaA, Darmstadt, Germany) was dissolved in double distilled water (dd${\text{H}}_{2}$O), to give a stock solution of 50-80 mg/mL. For the preparation of phantoms, all components, including a mixing tube, were placed on ice, and collagen (rat tail, ∼6 mg/mL in 0.01 M acetic acid, Merck KGaA, Darmstadt, Germany) was transferred to the mixing tube; CS and double distilled water were added to the collagen solution. Amounts of collagen and CS solution as well as final volumes are given in Table S1. To all mixture 0.15 mL 10x PBS were added, corresponding to approximately one tenth of the target final weight of the dried gel, giving phantoms with isotonic PBS concentration. Samples were thoroughly mixed and pH was adjusted to slightly basic (pH: 8-9) by drop-wise addition of 1 M sodium hydroxide (NaOH) and 0.5 M hydrochloric acid (HCl). The mixing tubes containing the collagen mixtures were placed into a warm water bath at 37 ^∘^C to solidify the mixture into a gel. The samples were initially kept in the bath for 45-60 minutes, after which the gels were checked for their solidity. If necessary, the pH adjustment and incubation was repeated. After the gels were solid, they were removed from the mixing tubes and transferred into a plastic cell culture dish, which was placed into a laminar flow oven at 40 ^∘^C. The dishes were covered with an inverted beaker to avoid contamination, while still allowing airflow from below. Drying continued until the mass of each sample (with one exception, see Table 1) was reduced to 1.50 ± 0.03 g, after which the samples were placed into small plastic tubes for MRI measurements. The tubes were sealed with a cap in order to prevent dehydration over time and stored refrigerated. For MRI measurements the phantoms were brought to room temperature; all samples were placed into the scanner simultaneously in a circular arrangement.

### 2.2 MRI measurements

The relaxation rates were determined at three different magnetic field ($B_{0}$) strengths. The samples were measured using 1.5 T (Siemens Magnetom Aera, Germany) and 3 T (Siemens Magnetom Aera, Germany) MRI scanners, in combination with a 15 channel transmit/receiver coil (QED, Mayfield Village, OH, USA) at Oulu University Hospital in a room equipped with air conditioning for temperature control. Three-dimensional images of the samples were obtained using a 3D proton density weighted (SPACE) sequence. $R_{1}$ values were measured using an inversion recovery turbo spin echo (IR TSE) sequence, while $R_{2}$ values were measured with a multi-echo spin echo sequence (MESE/MapIT). $R_{1\rho }$ relaxation rates were only measured at 9.4 T due to a lack of a suitable sequence for the Siemens MRI scanners at lower magnetic field strengths. Pulse sequence parameters used in the 1.5 and 3.0 T measurements are shown in Table S2 of the SI.

The measurements of $R_{1}$, $R_{2}$, and $R_{1\rho }$ at 9.4 T were performed at the University of Eastern Finland with a 9.4 T MRI scanner (Oxford instruments Plc, Witney, UK) in combination with a 19-mm quadrature volume RF transceiver (RAPID Biomedical GmbH, Rimpar, Germany) and VnmrJ3.1 Varian/Agilent DirectDrive console. The sequences used in the 9.4 T measurements were set to be as similar as possible to the ones used in the 1.5 T and 3.0 T measurements. Pulse sequence parameters used in the 9.4 T measurements are shown in Table S3 in SI.

Two to four of the most homogeneous slices were selected from the MR images by determining their locations from three-dimensional images, and appropriate regions of interest (ROI) were drawn on the slices using a Matlab-based program (Aedes, University of Eastern Finland, Kuopio, Finland, http://aedes.uef.fi). ROIs were drawn so that they were in similar locations for all three measurements by matching the positions of the multi-slice setups between different measurements. This was done by calculating the distance of the slice from the bottom of the phantom. In all measurements, $R_{1}$ and $R_{2}$ maps of the phantoms were calculated using in-house written plugins for Aedes, fitting image intensities voxel-wise to a mono-exponential signal model in order to create a two-dimensional relaxation time map. The final values were weighted averages over all non-zero segmented voxels. The mono-exponential signal model used for $R_{1}$ was

(1) $$S(t)=S_{0}(1-2\exp(-(\text{TI}\cdot R_{1})))$$

and the model for $R_{2}$ was

(2) $$S(t)=S_{0}\exp(-(\text{TE}\cdot R_{2})).$$

Here, $S_{0}$ is the intensity of the original signal, TI is the inversion time and TE is the echo time. In the 9.4 T measurements, $R_{1\rho }$ maps were created by making two-parameter fits of the raw data to Equation (3):

(3) $$S(t)=S_{0}\exp(-(\text{TSL}\cdot R_{1\rho }))$$

where TSL is the spin-lock time. Relaxation times were obtained from the maps within the previously determined ROIs and converted into relaxation rates using Aedes.

By performing a multiple linear regression estimation of the relaxation data, a prediction of the rates at concentrations comparable to cartilage was calculated. The model has two independent variables and the estimation was done with Python 3.9 using the scikit-learn package [16]. The model used in the extrapolations is shown in Equation (4):

(4) $$R=\beta_{0}+\beta_{col}c_{col}+\beta_{CS}c_{CS}$$

Here, $c_{col}$ and $c_{CS}$ are the collagen and CS concentrations, respectively, $\beta_{0}$ the intercept and $\beta_{col}$ and $\beta_{CS}$ the model coefficients for collagen and CS, respectively. For fits of $R_{2}$ and $R_{1\rho }$, $\beta_{0}$ was set to zero (i.e. not included in the fit), as for the full model *p*-values for all coefficients were larger than 0.05 and in some cases unphysical, negative values for $\beta_{0}$ were obtained. Also, an interaction term ($c_{col}\cdot c_{CS}$) was tested for all models, but found not to improve the model. To obtain an estimate of relaxation rates at physiological concentrations, the model was extrapolated to 185 mg/g collagen and 45 mg/g CS, based on the range of values given by estimate by Mow et al.[17], [17] and Maroudas et al.[18], [18] for articular cartilage. The value used for collagen corresponds to the mean of the range given by Mow et al., and the highest value found in the study by Maroudas et al.. The concentration of CS would correspond to a low total glycosaminoglycan (GAG) concentration in the study by Maroudas et al.. However, although Mow et al. only give a range of 4-7% for the total PG concentration, this range suggests that the CS concentration used here is more likely to over- than to underestimate the contribution of CS to the relaxation.

## 3 Results

**Figure 1:**
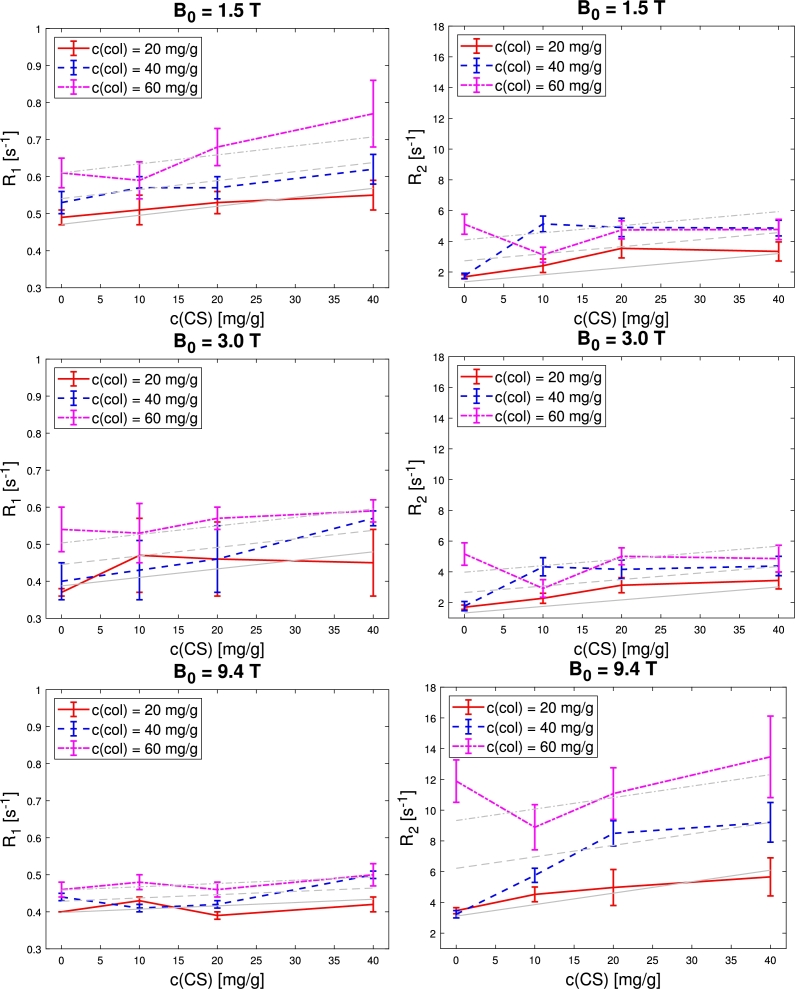
*R*_1_ (left), and *R*_2_ (right) values as a function of CS concentration at 1.5 T (top), 3.0 T (center), and 9.4 T (bottom). Error bars indicate standard deviations. Gray lines show the corresponding linear fit. Please note that error bars and slope can appear exaggerated to a shifted range of the y-axis.

As presented in Table 2 and Fig. 1, $R_{1}$ tend to increase with collagen concentration, as the 60 mg/g collagen concentration series had the highest relaxation rates at all magnetic fields, while the 20 and 40 mg/g series' values were similar. In the 9.4 T measurements the differences in $R_{1}$ values were the smallest. Similarly, $R_{1}$ increases with CS concentration in the sample series. Generally, $R_{1}$ relaxation rates decreased with increasing magnetic field strength (this trend is more clearly illustrated in the additional plots found in Fig. S1 in the Supporting Information). This effect was most distinct in the samples with highest collagen content.

**Table 2:** Mean values of *R*_1_ relaxation rates ± standard deviations [s^−1^].

B_0_	Collagen [mg/g]	CS [mg/g]			
		0	10	20	40
1.5 T	20	0.49 ± 0.02	0.51 ± 0.04	0.53 ± 0.03	0.55 ± 0.04
	40	0.53 ± 0.03	0.57 ± 0.03	0.57 ± 0.03	0.62 ± 0.04
	60	0.61 ± 0.04	0.59 ± 0.05	0.68 ± 0.05	0.77 ± 0.09

3.0 T	20	0.37 ± 0.01	0.47 ± 0.10	0.46 ± 0.10	0.45 ± 0.09
	40	0.40 ± 0.05	0.43 ± 0.08	0.46 ± 0.09	0.57 ± 0.02
	60	0.54 ± 0.06	0.53 ± 0.08	0.57 ± 0.03	0.59 ± 0.03

9.4 T	20	0.40 ± 0.00	0.43 ± 0.01	0.39 ± 0.01	0.42 ± 0.02
	40	0.44 ± 0.01	0.41 ± 0.01	0.42 ± 0.01	0.50 ± 0.01
	60	0.46 ± 0.02	0.48 ± 0.02	0.46 ± 0.02	0.50 ± 0.03

$R_{2}$ values (Table 3 and Fig. 1, as well as Fig. S1 in SI) show only weak, in parts non-significant, upwards trends with increasing collagen and CS concentrations at 1.5 T and 3.0 T. Measured values of $R_{2}$ were significantly higher (roughly twice as high) at 9.4 T compared to the lower magnetic fields, as illustrated by Figs. 1 and S1. At 9.4 T, especially the $R_{2}$ values of the 60 mg/g collagen concentration series were noticeably higher compared to lower concentrations, contributing to an overall clear trend towards higher $R_{2}$ with increasing collagen concentration. This is most distinctively observed for the samples with c(CS)=10mg/g that only at 9.4 T show a monotonous increase in $R_{2}$ with collagen concentration. At the same time, this qualitative difference between the series at 9.4 T and lower field strengths could also be due the comparatively high $R_{2}$ values observed for the phantom with c(collagen)=40mg/g and c(CS)=10mg/g at the two lower field strengths.

**Table 3:** Mean values of *R*_2_ relaxation rates ± standard deviations [s^−1^].

B_0_	Collagen [mg/g]	CS [mg/g]			
		0	10	20	40
1.5 T	20	1.69 ± 0.14	2.41 ± 0.44	3.54 ± 0.62	3.34 ± 0.62
	40	1.75 ± 0.17	5.13 ± 0.51	4.90 ± 0.60	4.86 ± 0.51
	60	5.11 ± 0.65	3.12 ± 0.49	4.74 ± 0.59	4.77 ± 0.66

3.0 T	20	1.70 ± 0.14	2.28 ± 0.33	3.13 ± 0.49	3.44 ± 0.55
	40	1.77 ± 0.30	4.33 ± 0.59	4.16 ± 0.61	4.38 ± 0.63
	60	5.16 ± 0.73	2.92 ± 0.57	5.01 ± 0.55	4.86 ± 0.87

9.4 T	20	3.46 ± 0.20	4.52 ± 0.48	4.97 ± 1.17	5.66 ± 1.24
	40	3.23 ± 0.24	5.76 ± 0.46	8.49 ± 0.82	9.21 ± 1.29
	60	11.89 ± 1.38	8.89 ± 1.47	11.08 ± 1.68	13.47 ± 2.65

**Figure 2:**
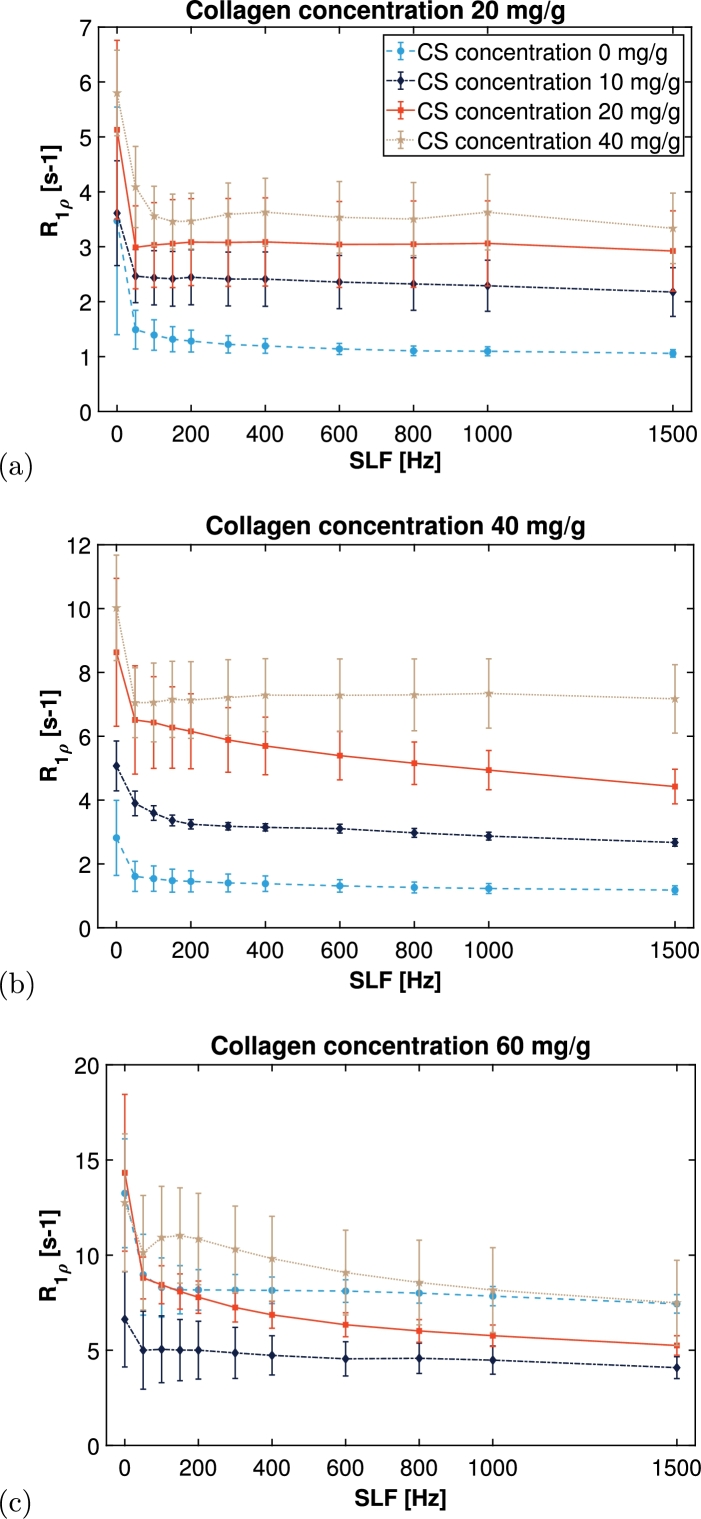
*R*_1*ρ*_ relaxation rates of the different sample series as a function of spin-lock frequency (SLF) with collagen concentration being (a) 20 mg/g, (b) 40 mg/g, and (c) 60 mg/g. Error bars indicate standard deviations.

$R_{1\rho }$ increases both with increasing collagen (Figure S2 in SI) and CS concentration as shown in Fig. 2. The only exception to this was the 60 mg/g collagen sample without CS (Fig. 2c), which had higher $R_{1\rho }$ values than some of the higher CS concentration samples. Otherwise, the changes in $R_{1\rho }$ values with CS concentration also increased with collagen concentration. The spin-lock frequency (SLF) discernibly affects $R_{1\rho }$ relaxation rates only at low SLF frequencies, i.e. below 100 Hz.

Using a multiple linear regression estimation of the relaxation data shown in Equation (4), relaxation rates were extrapolated to concentrations closer to ones in cartilage. The estimates of $R_{1}$ and $R_{2}$ with the model parameters and R-squared values of the fits are shown in Table 4. With the coefficient for collagen being consistently higher than for CS, and the target concentration of collagen nearly four times that of CS, the contribution of collagen to all extrapolated relaxation rates is at least five times larger than the contribution from CS. The $R_{1}$ extrapolation of the 1.5 T data of 1.16 ${\text{s}}^{-1}$ ($T_{1}$ = 866 ms) would be quite close to some of the values of in vivo cartilage $T_{1}$ at 1.5 T, with values ranging from 400-1100 ms varying with cartilage depth [17], [18], [18], [17], [19]. The $R_{2}$ extrapolation of 32.11 ${\text{s}}^{-1}$ ($T_{2}$ = 31 ms) was also in the range of measured $T_{2}$ values at 9.4 T, between 22-62 ms [20]. While it is established that the relaxation observed in cartilage is strongly dependent on its structure, and the present findings must be interpreted with caution, since the extrapolation is done from 12 data points that are far from the extrapolated values, these results imply that the overall relaxation rates also can be explained based in terms of the chemical composition. The $R_{2}$ fits at 1.5 T is relatively poor with the lower end of the 95% confidence interval of $\beta_{CS}$ being negative (for the coefficient also p>0.05, cf. SI Table S4). The intercepts of the $R_{2}$ and $R_{1\rho }$ regression models being set to zero reveal a limitation in our data and/or model, since this would imply $R_{1}$ > $R_{2}$ at small concentrations.

**Table 4:** Results of the linear regression model [Eq. (4)] for the various relaxation rates at different magnetic field strengths, including model parameters (*β*_*x*_) and 95% confidence interval given in parentheses. Corresponding *p*-values are found in Supporting Information Table S4. Predicted values for relaxation rates and times at physiological concentrations are calculated using *c*_*col*_ = 185 mg/g and *c*_*CS*_ = 45 mg/g.

Series	*B*_0_ [T]	Fitting parameters			Predicted values	
		*β*_0_	*β*_*col*_	*β*_*CS*_	*R*_*x*_ [s^−1^]	*T*_*x*_ [ms]
*R*_1_	1.5	0.4019	0.0035	0.0024	1.16	866
		(0.3567, 0.4472)	(0.0025, 0.0044)	(0.0014, 0.0035)		
	3.0	0.3295	0.0029	0.0023	0.97	1032
		(0.2580, 0.4009)	(0.0014, 0.0044)	(0.0006, 0.0040)		
	9.4	0.3681	0.0015	0.0009	0.688	1455
		(0.3277, 0.4085)	(0.0006, 0.0024)	(0.0000, 0.0018)		

*R*_2_	1.5		0.0683	0.0459	14.69	68.06
			(0.0426, 0.0939)	(-0.0020, 0.0937)		
	3.0		0.0663	0.0424	14.18	70.54
			(0.0454, 0.0873)	(0.0033, 0.0814)		
	9.4		0.1554	0.0747	32.11	31.14
			(0.1257, 0.1851)	(0.0192, 0.1301)		

*R*_1*ρ*_ (600 Hz)	9.4		0.0878	0.0682	19.31	51.79
			(0.0590, 0.1165)	(0.0146, 0.1218)		

## 4 Discussion

$R_{1}$ values generally increased with increasing collagen concentration at all measured magnetic fields. $R_{1}$ also decreased with increasing magnetic field strength, as predicted by relaxation theories and experimentally observed previously [21], [22], [23], [24], [25]. Relative differences in $R_{1}$ values between low and high collagen concentrations decreased with increasing magnetic field strength, suggesting that $R_{1}$ values are more sensitive to collagen concentration at lower magnetic fields.

$R_{2}$ values generally increased with increasing collagen concentration. At low collagen concentrations, also an increase in $R_{2}$ values with increasing CS concentration was observed, suggesting that contributions from collagen to relaxation already at intermediate concentrations became dominant enough to mask the contribution of CS. This would certainly be true in articular cartilage, in which the amount of collagen is significantly higher than that of CS [26]. The concentrations of either collagen or CS in our samples were higher than in most of the previous collagen phantom studies. Still, collagen concentrations in our phantoms (20-60 mg/g) were significantly below the levels that naturally occur in cartilage.

The unexpected increase of $R_{2}$ values at 9.4 T could suggest that the $R_{2}$ measurements are not directly comparable to each other, since different equipment and sequences were used. In collagen and CS, hydrogen atoms appear in multiple chemical groups, each of which have different motional characteristics affecting the relaxation via the dipole-dipole mechanism. As each $B_{0}$ field strength weights the motions differently, according to their correlation times, it is not unexpected to find the $B_{0}$-dependence of relaxation to be non-trivial. It should be noted that the $R_{2}$ TSE measurements were effectively the same as $R_{1\rho }$ measurements with SLF = 0 Hz, since the $R_{1\rho }$ pulse sequence that was used contained a refocusing pulse in the middle of the spin-lock sequence [27]. In contrast to $R_{1}$, the variation in $R_{2}$ values increases with increasing magnetic field strength. This suggests $R_{2}$ to be more sensitive to concentration changes of the different components compared to $R_{1}$ at higher $B_{0}$.

In our experiments, $R_{1\rho }$ values increase with increasing collagen and CS concentrations in nearly all sample series at 9.4 T. A dispersive effect in $R_{1\rho }$ could only be seen at low spin-lock frequencies below 100 Hz. The only series exhibiting a clear effect of the spin-lock pulse between 100 Hz and 1.5 kHz is the 60 mg/g collagen concentration series. A dispersive effect like the one previously described for some *ex vivo* cartilage $T_{1\rho }$ experiments, [28], [29] is missing in our gel samples with lower concentrations. This suggests that a dispersion at higher frequencies might be visible at higher collagen concentrations, closer to the ones in articular cartilage. However, as the aforementioned studies used different spin-lock frequencies, certain differences are to be expected from our study. Still, a possible reason for this could be that the range of spin-lock frequencies used here is too narrow to elicit dispersion. In any case, a dispersive effect at low frequencies that could be accessible in (clinical) *in vivo* measurements could be observed here. The quality and applicability of the effects produced by spin-lock pulses at low frequencies, however, still need to be investigated further.

The collagen concentrations of the phantoms were raised significantly from the stock concentration of the collagen solution by drying them in a laminar flow oven. Orientation of the collagen fibers was assumed to be isotropic in the gel. Fiber orientation has been shown in previous studies to influence the appearance of cartilage samples in MR images and the relaxation properties of articular cartilage [30], [31]. In our study, however, the isotropic phantoms enabled us to separately assess the role of orientation-independent mechanisms, since we were interested in the purely concentration-dependent effects on relaxation. Some of the samples had issues with gel uniformity, as air bubbles were inadvertently created in the samples during mixing. Also, the composition and homogeneity of the samples was not verified after drying. Due to the lack of a suitable sequence at lower magnetic fields, $R_{1\rho }$ values were only obtained at 9.4 T, meaning a comparison between $R_{1\rho }$ values at different $B_{0}$ could not be done.

## 5 Conclusions

In this study, we created hydrogel MRI phantoms that only contained the most important macromolecular components of the biological hydrogel articular cartilage, collagen and chondroitin sulfate (CS), in order to understand the contributions to relaxation on a molecular level. In the collagen gel phantoms, $R_{1}$ values generally increased with both collagen and CS concentration at magnetic fields commonly used in clinical MRI, like 1.5 T and 3.0 T. At a high magnetic field of 9.4 T, $R_{2}$ values also showed an increase with both collagen and chondroitin sulfate concentrations. At this field strength, also $R_{1\rho }$ values were found to depend on both collagen and CS content, increasing with the concentration of either substance, although spin-lock frequency had a limited effect on the values. Extrapolation of our data suggests that both collagen and CS concentrations affect the $R_{1}$ and $R_{2}$ values of cartilage, with the contribution of collagen clearly dominating. A linear model could also be used to extrapolate values into physiological concentrations from relatively low concentrations. This implies that large part of the total relaxation rates observed in articular cartilage can be explained by its chemical composition, even though it is known that relaxation rates – certainly $R_{2}$ – are highly sensitive to its structural properties.

## Funding

This work was supported by the 10.13039/501100004012Jane and Aatos Erkko Foundation; the 10.13039/501100002341Academy of Finland [project numbers 285909 and 297033];and the 10.13039/501100003125Finnish Cultural Foundation.

## CRediT authorship contribution statement

**Olli-Pekka Aro:** Writing – original draft, Visualization, Methodology, Investigation, Formal analysis, Data curation, Conceptualization. **Victor Casula:** Writing – review & editing, Validation, Supervision, Project administration, Methodology, Conceptualization. **Nina E. Hänninen:** Writing – review & editing, Validation, Methodology, Investigation. **Jouni Karjalainen:** Writing – review & editing, Validation, Data curation. **Mikko J. Nissi:** Writing – review & editing, Validation, Supervision, Methodology, Data curation. **Miika T. Nieminen:** Writing – review & editing, Resources, Project administration, Funding acquisition. **Henning Henschel:** Writing – review & editing, Visualization, Validation, Supervision, Project administration, Methodology, Conceptualization.

## Declaration of Competing Interest

The authors declare that they have no known competing financial interests or personal relationships that could have appeared to influence the work reported in this paper.

## Appendix A Supplementary material

MMC The supplementary information contains additional details on phantom composition, used pulse sequences, and statistical parameters for the regression model, as well as additional plots of the relxataion rates. MMC
