# Supplementary material for: Effects of collagen and chondroitin sulfate on relaxation at multiple magnetic field strengths
Source: Heliyon. 2025 Jan 13;11(2):e41854. doi: 10.1016/j.heliyon.2025.e41854 (PMC11783438; doi:10.1016/j.heliyon.2025.e41854)
Supplement: MMC — The supplementary information contains additional details on phantom composition, used pulse sequences, and statistical parameters for the regression model, as well as additional plots of the relxataion rates. [file mmc1.pdf]

# – Supporting Information – Effects of collagen and chondroitin sulfate on relaxation at multiple magnetic field strengths

Olli-Pekka Aro[1], Victor Casula[1], Nina E. Hänninen[1,2], Jouni Karjalainen[1], Mikko J. Nissi[1,2], Miika T. Nieminen[1,3], and Henning Henschel[1,4,*]

[1]Research Unit of Health Sciences and Technology, University of Oulu, Oulu, Finland
[2]Department of Technical Physics, University of Eastern Finland, Kuopio, Finland
[3]Department of Diagnostic Radiology, Oulu University Hospital, Finland
[4]Department of Medicinal Chemistry, Uppsala University, Husargatan 3, Box 574, SE-75123 Uppsala, Sweden.
[*]Corresponding author: henning.henschel@ilk.uu.se

Contents

S1 Collagen gel contents and pulse sequence parameters **S2**

S2 Additional plots of sample relaxation values **S5**

S3 *p*-values of the regression model **S6**

S1 Collagen gel contents and pulse sequence parameters

Table S1: Nominal concentrations (c_{nom}) and volumes of stock solutions (V_s) used in sample preparation. Values marked with an asterisk (*) used a CS solution with a stock concentration of 50 mg/ml, while all others used a solution of 80 mg/ml.

Sample no.	Collagen		CS		Total Volume
	c_{nom} [mg/g]	V_s [mL]	c_{nom} [mg/g]	V_s [mL]	[mL]
1	20	5	0	0	6
2	20	5	10	0.1875	6
3	20	5	20	0.375	6
4	20	5	40	0.750	6
5	40	10	0	0	11
6	40	10	10	0.300*	11
7	40	10	20	0.600*	11
8	40	10	40	0.750	11
9	60	15	0	0	16
10	60	15	10	0.1875	16
11	60	15	20	0.375	16
12	60	15	40	0.750	16

Table S2: Pulse sequence parameters for the 1.5 T and 3.0 T measurements.

**Sequence**	**Parameters**
**3D PD SPACE**	Resolution = 0.5x0.5x0.5 mm^3<br>TR = 1200 ms<br>TE = 26 ms<br>Flip angle = 120°<br>No. of slices = 72<br>No. of averages = 2<br>ETL = 55<br>BW = 425 Hz/pixel
**T1 IR TSE**	Resolution = 0.4x0.4x2.0 mm^3<br>TR = 10000 ms<br>TI = 400, 800, 1600, 3200, 7000, 9000 ms<br>ESP = 4 ms<br>ETL = 8 with centric echo ordering<br>No. of slices: = 7<br>No. of averages = 2<br>Interslice gap = 1 mm
**T2 MESE**	Resolution = 0.4x0.4x2.0 mm^3<br>TR = 10000 ms<br>TE = 12, 24, 36, 48, 60, 72, 84, 96, 108, 120, 132, 144, 156, 168, 180, 192, 250, 300, 350, 400, 450, 500, 550, 600, 650, 700, 750, 800 ms<br>ESP = 4.3 ms<br>ETL = 16 with centric echo ordering<br>No. of slices = 7<br>No. of averages = 2<br>Interslice gap = 1 mm

PD = proton density, ESP = echo spacing, ETL = echo train length, MESE = multi-echo spin-echo, TSE = turbo spin-echo, TI = inversion time, TE = echo time, TR = repetition time, TSL = spin-lock time.

Table S3: Pulse sequence parameters for the 9.4 T measurements.

Sequence	Parameters
**T1 IR TSE**	Resolution = 0.4x0.4x2.0 mm^3<br>TR = 10000 ms<br>TI = 400, 800, 1600, 3200,<br>5000, 7000 and 9000 ms<br>ESP = 5 ms<br>ETL = 8 with centric echo ordering<br>No. of averages = 1<br>Interslice gap = 1 mm
**T2 MESE**	Resolution = 0.4x0.4x2.0 mm^3<br>TR = 10000 ms<br>TE = 12-768 ms, in increments of 12 ms<br>ESP = 5 ms<br>ETL = 8 with centric echo ordering<br>No. of averages = 1<br>Interslice gap = 1 mm
**T1ρ TSE**	Resolution = 0.4x0.4x2.0 mm^3<br>TR = 10000 ms<br>TSL = 0, 10, 20, 40, 60,<br>80, 100, 200 and 400 ms<br>ESP = 5 ms<br>ETL = 8 with centric echo ordering<br>No. of averages = 1<br>Interslice gap = 1 mm<br>SLF = 50, 100, 150, 200, 300,<br>400, 600, 800, 1000, 1500 Hz

ESP = echo spacing, ETL = echo train length, MESE = multi-echo spin-echo, TSE = turbo spin-echo, TI = inversion time, TE = echo time, TSL = spin-lock time.

S2 Additional plots of sample relaxation values

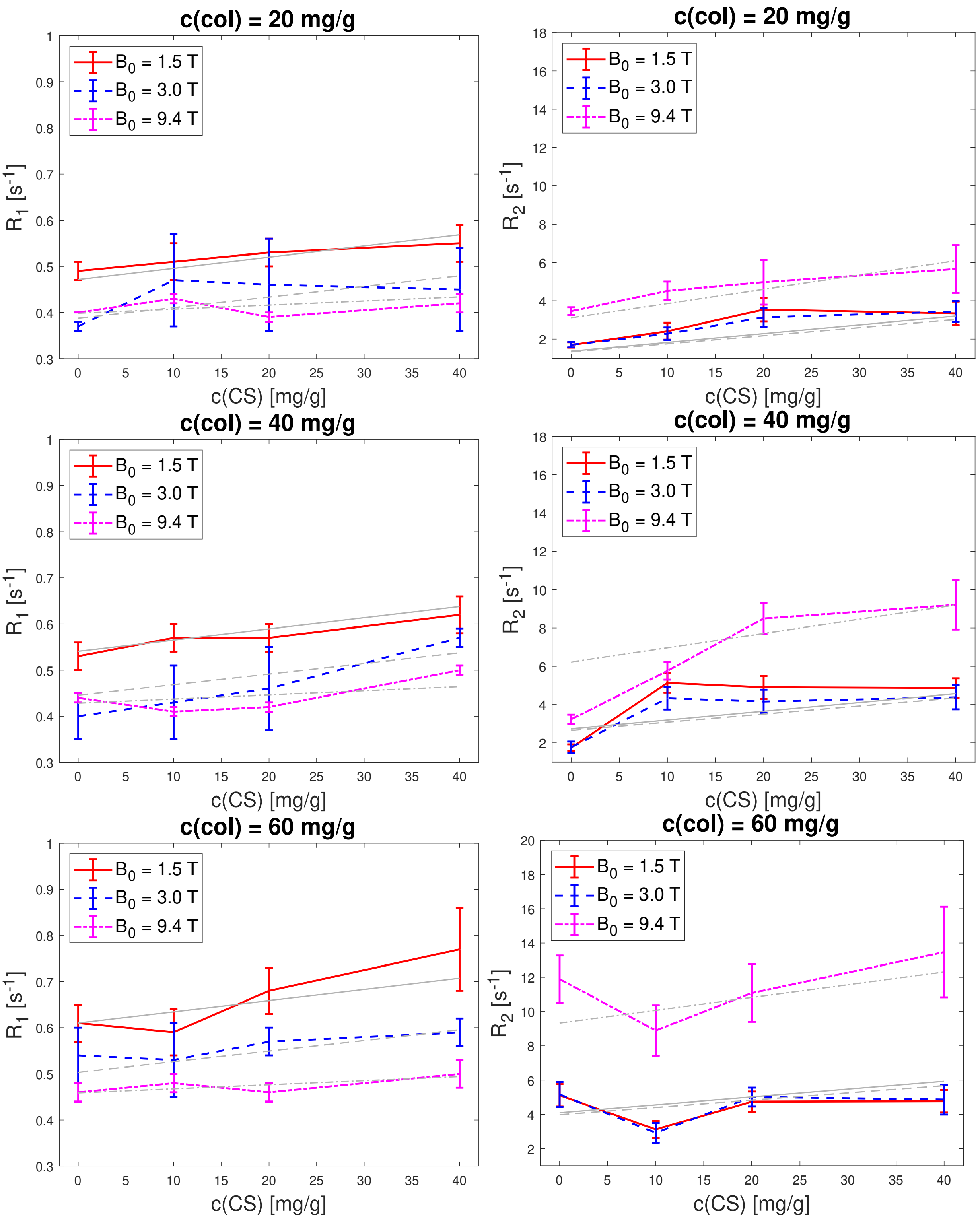

Figure S1: R_1 (left), and R_2 (right) values shown as a function of CS concentration with collagen concentration being 20 mg/g (top), 40 mg/g (center), and 60 mg/g (bottom). Error bars indicate standard deviations. Grey lines show the corresponding linear fit.

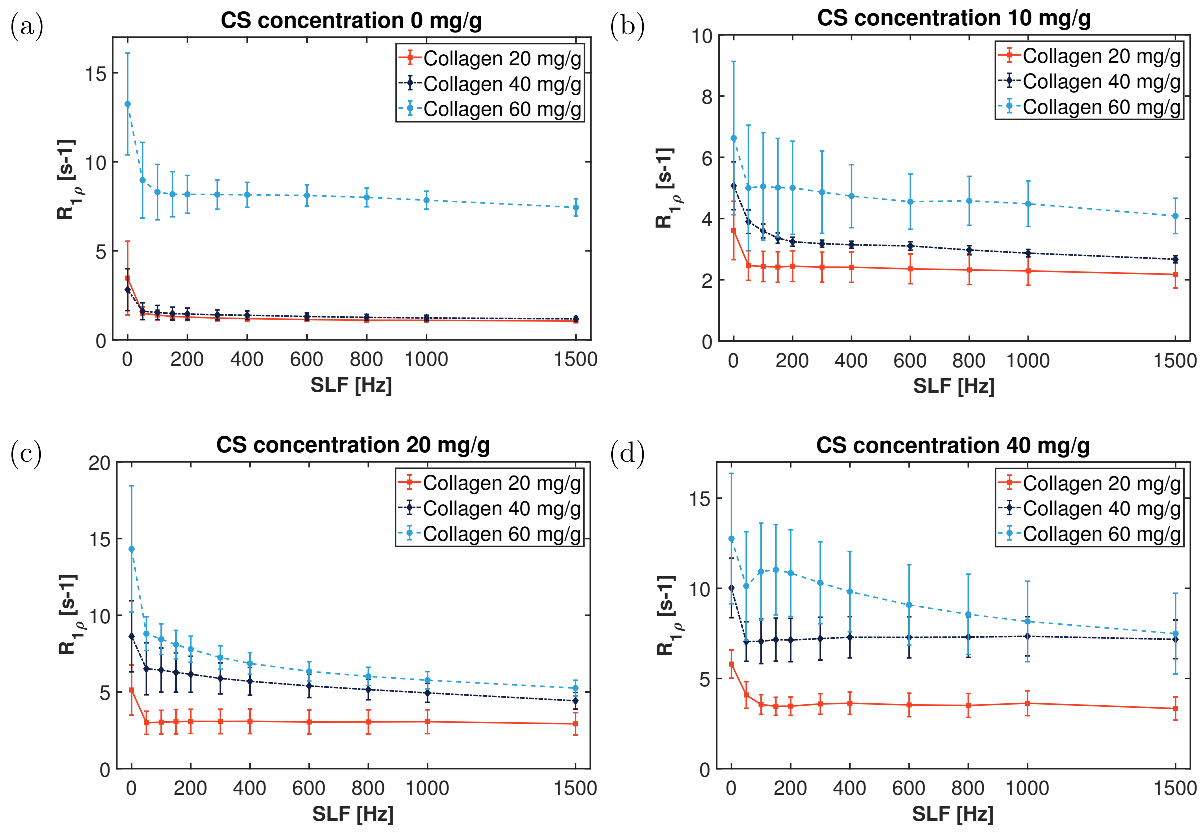

Figure S2: $R_{1\rho}$ relaxation rates of the different CS concentrations as a function of spin-lock frequency (SLF) with CS concentration being 0 mg/g (a), 10 mg/g (b), 20 mg/g (c), and 40 mg/g (d). Error bars indicate standard deviations.

S3 *p*-values of the regression model

Table S4: p-values of the linear regression model for the various relaxation rates at different magnetic field strengths

Series	B_0 [T]	Fitting parameters		
		$p(\beta_0)$	$p(\beta_{col})$	$p(\beta_{CS})$
R_1	1.5	$8.7 \cdot 10^{-9}$	$2.0 \cdot 10^{-5}$	$6.1 \cdot 10^{-4}$
	3.0	$2.5 \cdot 10^{-6}$	$2.1 \cdot 10^{-3}$	0.013
	9.4	$6.9 \cdot 10^{-9}$	$3.3 \cdot 10^{-3}$	0.063
R_2	1.5		$1.5 \cdot 10^{-4}$	0.058
	3.0		$3.4 \cdot 10^{-5}$	0.036
	9.4		$3.9 \cdot 10^{-7}$	0.013
$R_{1\rho}$ (600 Hz)	9.4		$4.7 \cdot 10^{-5}$	0.018
